# Complement and Complement Targeting Therapies in Glomerular Diseases

**DOI:** 10.3390/ijms20246336

**Published:** 2019-12-16

**Authors:** Sofia Andrighetto, Jeremy Leventhal, Gianluigi Zaza, Paolo Cravedi

**Affiliations:** 1 Department of Medicine, Division of Nephrology, Icahn School of Medicine at Mount Sinai, 1 Levy Place, New York, NY 10029, USA; sofia.andrighetto@gmail.com (S.A.); jeremy.leventhal@mssm.edu (J.L.); 2 Renal Unit, Department of Medicine, University/Hospital of Verona, 37126 Verona, Italy; gianluigi.zaza@univr.it

**Keywords:** complement, alternative complement pathway, complement-targeting therapies, C3 glomerulopathy, hemolytic uremic syndrome, focal segmental glomerulosclerosis

## Abstract

The complement cascade is part of the innate immune system whose actions protect hosts from pathogens. Recent research shows complement involvement in a wide spectrum of renal disease pathogenesis including antibody-related glomerulopathies and non-antibody-mediated kidney diseases, such as C3 glomerular disease, atypical hemolytic uremic syndrome, and focal segmental glomerulosclerosis. A pivotal role in renal pathogenesis makes targeting complement activation an attractive therapeutic strategy. Over the last decade, a growing number of anti-complement agents have been developed; some are approved for clinical use and many others are in the pipeline. Herein, we review the pathways of complement activation and regulation, illustrate its role instigating or amplifying glomerular injury, and discuss the most promising novel complement-targeting therapies.

## 1. Complement Cascade

The complement system consists of soluble or membrane-bound molecules, mostly zymogens, activated through a tightly regulated proteolytic cascade [1,2]. Current understanding of complement immune mechanisms include (1) functioning as opsonins; (2) producing chemoattractants to recruit immune cells thereby enhancing site specific angiogenesis, vasodilation and coagulation cascade regulator; and (3) functioning as an enhancing bridge to adaptive T and B lymphocyte responses [3]. Current research suggests that abnormal complement activation plays a role in autoimmune inflammatory diseases and particularly in those targeting the kidney.

## 2. Complement Cascade Activation and Regulation

**Figure 1:**
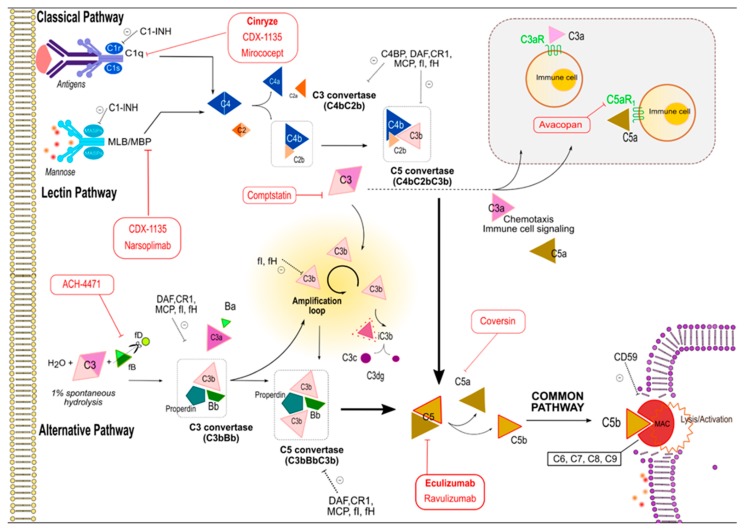
Overview of the complement cascade and principal complement targeting molecules. Three pathways can initiate complement cascade: (1) The classical, (2) the mannose-binding lectin (MBL), and (3) the alternative pathway. They all converge on C3 convertases formation which continuously cleave C3; after they are activated, the C3 convertase from alternative pathway dominates within an amplification loop that sustains the production of C3b (circular arrow). The three C3 convertases associate with an additional C3b to form the C5 convertases, which cleave C5 into C5a + C5b. C5b fragments recruits C6, C7, C8, and multiple C9 molecules to generate the terminal membrane attack complex (MAC); MAC inserts pores into cell membranes to induce cell lysis or activation. Anaphilotoxins C3a and C5a through their G protein-coupled receptors C3aR and C5aR, respectively, can promote signaling, inflammation, chemotaxis of leukocytes, vasodilation, cytokine and chemokine release, and activation of adaptive immunity. Dotted black arrows: inhibitor function of a complement effector on its target. Red arrows emerging from balloons: inhibitor function of anti complement drug on its target (bold drugs’ names are the FDA approved ones). Full arrow: consequential interaction between complement fractions leads to the subsequent cascade step. Full bold arrow: final convergence of the three complement pathways on same final target. MBL: Mannose binding lectin; MASP: Mannose-binding lectin-associated serine protease; C4BP: C4 binding protein; C1-INH: C1 inhibitor; DAF: Decay accelerating factor; CR1: Surface complement receptor 1; MCP: Membrane cofactor protein; CD59: Protectin; fD: Factor D; fB: Factor B; fI: Factor I; fH: Factor H. Red balloons highlights complement target drugs’ points of action (see Table 1).

Complement activation proceeds via three pathways: the classical, alternative, and mannitol-binding lectin (MBL). The pathways have both unique and overlapping proteins, activated by a proteolytic cascade, that respond to pathogenic insults [3,4] (Figure 1). The classical and MBL pathways are initiated by antibodies and bacterial mannose motifs binding to C1q and mannose-associated serine proteases (MASPs), respectively [5,6]. Conversely, spontaneous hydrolysis of C3 on cell surfaces produces constitutive alternative pathway (AP) activation [7], and is tightly controlled by a number of regulators [8]. Decay accelerating factor (DAF) and membrane cofactor protein (MCP) (CD46, murine homolog Crry) are cell surface-expressed complement regulators that accelerate the decay of surface-assembled C3 convertases, thereby limiting amplification of the downstream cascade. DAF restrains convertase-mediated C3 cleavage; MCP and factor H (fH) also have a cofactor activity: together with soluble factor I (fI), they irreversibly cleave C3b into iC3b, thereby preventing reformation of the C3 convertase.

The three activation pathways converge in C3 convertases that continuously cleave C3 into C3a and C3b. C3a signals on cell surface G protein-coupled receptor (GPCR) C3aR, while C3b forms additional alternative pathways C3 convertases (even if initiated via the other pathways), as well as C5 convertases. C5 convertases produce the split products C5a and C5b. While C5a functions similarly to C3a (but signals via C5aR) C5b, in conjunction with C6–C9, forms the membrane attack complex (MAC) leading to cell lysis/activation [9]. Countering distal complement MAC formation is CD59, a circulating complement inhibitor.

## 3. Effector Functions

Complement proteins promote inflammation and immune cell activity in multiple ways. C3a and C5a ligate their transmembrane-spanning receptors, C3aR and C5aR, on immune cells, leading to the production of proinflammatory cytokines and chemokines and promoting vasodilation. They also mediate neutrophil and macrophage chemoattraction, activate macrophages to promote intracellular killing of engulfed organisms, and contribute to T-cell and antigen-presenting cell (APC) activation, expansion, and survival [10,11,12,13]. The more distal complement proteins (C5b-9) form MAC complexes on cell membranes, promoting lysis of non-nucleated cells, such as red blood cells, or pathogens lacking cell surface complement inhibitors, such as bacteria. MAC insertion into nucleated eukaryotic cells generally does not result in lysis, but rather induces immune activation [14] and/or promotes tissue injury [15]. C3b and other bound cleavage products function as opsonins binding to specific surface-expressed receptors (complement receptors CR1, CR2, CR3, and CR4).

## 4. Complement in Glomerular Diseases

### 4.1. Diseases with Antibody-Mediated Complement Activation

The majority of circulating, or fluid-phase, complement components are produced by the liver. Liver produced complement components are involved in auto-antibody initiated glomerulonephritis (GN) through the classical and/or MBL pathways. Inadequate regulation of alternative pathway activity, due to inherited and/or acquired abnormalities of complement regulators, can result in glomerular injury from persistent C3 convertase activity with consequent excessive MAC activity. Complement can also be produced by parenchymal (e.g., tubular cells in the kidney [16] and resident/infiltrating immune cells, (e.g., T cells and APCs) [17,18,19]. The relative contributions of systemic or locally produced complement in GN pathogenesis remains unclear.

### 4.2. IgA Nephropathy

IgA nephropathy (IgAN) is the most common form of GN worldwide [20,21]; its clinical presentation varies, but it often includes proteinuria and hematuria. The disease is associated with aberrant O-glycosylation of mucosal IgA1 with galactose-deficient IgA1 (Gd-IgA1) which plays a pivotal role in the progression of IgAN [22]. They deposit in glomeruli and, subsequently, development of circulating/in situ immunoglobulins (IgA or IgG) targeting glycosylated IgA takes place [23,24]. Mesangial IgA and immune complexes deposits are often observed and initiate glomerular injury.

In vitro and in vivo studies showed that polymeric mucosal IgA activates the complement system through the alternative or MBL pathway [25], and glomerular MBL correlates with greater disease severity and a worse prognosis [26]. Although C3 levels in plasma are usually normal, glomerular C3 deposits can be detected in approximately 85% of biopsies, with C5b-9 also often present along with C3 during infections [27]. MAC generated from complement activation attack on mesangial cells inducing them to proliferate and over-produce oxidants, proteases, cytokines, growth factors (e.g., transforming growth factor β and platelet-derived growth factor) and extracellular matrix material that together result in the typical focal proliferative GN with mesangial matrix expansion characteristic of IgA nephropathy [28].

A genomic wide association study (GWAS) of IgAN in a cohort of 3144 cases of Chinese and European ancestry, linked allele deletion polymorphisms of complement factor H-related proteins one and three (*CFHR1* and *CFHR3*) with less severe IgA nephropathy [29,30]. Since CFHR proteins interfere with factor H complement regulation (Figure 1) their deficiency might reduce complement activation and lessen IgAN. CFHR1, CFHR3, and CFHR5 especially have been studied for their role in IgAN [29,31]; another study of 1126 Chinese patients concluded that circulating CFHR5 levels are an independent risk factor for the disease: levels were higher in IgAN subjects compared to heathy controls, and correlated directly with worst Oxford MEST pathology score, a lower glomerular filtration rate (GFR), and hypertension [32].

### 4.3. Membranous Nephropathy

Membranous nephropathy (MN) is the second most commonly diagnosed GN, and the most common cause for nephrotic syndrome in adults (20–50%) [33]. Disease progression varies greatly with ~1/3 of patients undergoing spontaneous remission, ~1/3 developing progressive renal insufficiency, and ~1/3 maintaining normal GFR despite persistent proteinuria. MN is characterized by presence of anti-podocyte antibodies in the subepithelial space of glomerular capillary loops and granular deposits of IgG4 and C3. M-type phospholipase A2 receptor (PLA_2_R) has been found as the main target podocyte antigen for autoantibodies in 70–80% of patients with primary MN, while antibodies against thrombospondin type 1 domain-containing 7A (THSD7A) are detected in a minority of patients [34]. Although antibodies from IgG4 subclass are poor classical complement pathway activators, deposition of C3 and breakdown of C4b products are detectable in almost all patients with a primary form of MN [27]. Mannose binding lectin and MBL-associated serine protease expression (MASP-1, MASP-2) are detected in PLA_2_R positive patients’ glomeruli, suggesting that complement activation proceeds through this pathway [35]. Hypogalactosylated IgG (including IgG4) binds MBL and activates the complement providing a possible explanation to the IgG4 conundrum [36]. Animal studies also indicate a role for MAC insertion into podocytes; blocking their formation prevents disease [37]. Sublytic activation alters podocyte cytoskeletal structure crucial for slit diaphragm integrity and function, leading to proteinuria [38,39].

### 4.4. Post Infectious Glomerulonephritis

Post infectious GN is a common cause of nephritic syndrome that develops after self-limited bacterial infections (most commonly from *streptococcal* or *staphylococcal* species). It occurs mainly in childhood, but can also be seen in adults. It is characterized by hypercellularity within the capillary loops (caused by neutrophils infiltration and endothelial proliferation) and strong C3 staining, usually in addition to IgG. Post infectious GN occurs due to passive glomerular trapping of circulating immune complexes composed of nephritogenic bacterial antigens and IgG, complement activation, and attraction of neutrophils responsible for glomerular injury [28]. However, levels of C1q and C4 deposition are lacking or low in most of the cases [40,41], suggesting contributions from lectin and alternative pathway. This is eventually triggered from specific pathogens’ components; for example, streptococcal pyrogenic exotoxin B is a possible alternative pathway activation [42]. Autoantibodies with C3 nephritic factor (C3nef), activity that binds to and stabilizes C3 convertases, has also been reported in post-infectious GN and may be associated with an enhanced cleavage of C3 [28]. In some patients underlying genetic defects in the regulation of the alternative pathway, including mutations in complement regulators (fH or CFHR5) and presence of C3Nef, lead to persistent glomerular deposition of complement factors within the glomeruli and inflammatory infiltrates that resemble features of a persistent proliferative glomerulonephritis [43]. Interestingly, in few cases, post infectious GN evolved into C3 glomerulopathy (C3G) [44]: recent reports document repeat biopsies demonstrating transformation of post infectious GN to C3G, including identical appearing early lesions of C3G and initiation of C3G by streptococcal infection. Sethi et al. [43] described that most of the cases with biopsy-proven persistent post-infectious GN had underlying genetic mutations and/or auto-antibodies affecting regulation of the alternative complement pathway. These findings indicate that glomerular injuries initiated by infection may transfer to C3G by imbalanced alternative complement pathway activation: C3G is initiated by heterogeneous insults, leading to a final common pathway of alternative complement dysregulation.

### 4.5. Immune Complex-Mediated Membranoproliferative Glomerulonephritis (MPGN)

Membranoproliferative glomerulonephritis (MPGN) is a histopathological pattern of glomerular injury characterized by mesangial hypercellularity, capillary wall changes (i.e., “tram-tracking”), and endocapillary proliferation found in 7–10% of biopsy-diagnosed glomerulonephritis [45]. MPGN classification was based on electron micrograph ultrastructural findings but advances in our understanding of underlying pathomechanisms produced a rethinking of MPGN and a classification schema based on immunofluorescence findings; MPGN is caused by immune complex deposition, C3 dysregulation, or thrombotic microangiopathy (TMA) [45]. Immune complex-mediated MPGN is caused by immune complex deposition in the subendothelial space activating complement classical pathways and causing glomerular injury. When not linked to a systemic disease, it is termed ‘idiopathic’ but secondary forms more commonly occur in association with infections (e.g., hepatitis B, C, or tuberculosis), autoimmune diseases (e.g., Sjogren’s Syndrome or systemic lupus erythematosus SLE), or monoclonal gammopathy. Clinical evidence of classical complement activation in immune complex-mediated MPGN includes preferential consumption of plasma C4 (although C3 is often low as well) and detection of C1q and terminal C5b-9 complex in glomeruli. This phase is followed by an influx of leukocytes, promoted by formation of the C3a and C5a anaphylatoxins, leading to capillary damage and proteinuria [46]. Activation of classical pathway through immunoglobulins is the most prominent pathogenic process, but heterozygous mutations in alternative pathway complement regulators and the presence of circulating C3nef factor are also identified in some patients with immune complex-mediated MPGN, suggesting additional contributions from the alternative pathway [47]. These findings raise the possibility that in individuals with genetic or acquired complement alternative pathway dysregulation, immune complex deposition initially triggers injury through the classical pathway but chronic kidney injury is sustained through the enhanced alternative pathway [46]. The complement also features prominently in the two other dominant etiologies of MPGN: C3 glomerulopathies and TMA from atypical Hemolytic Uremic Syndrome (aHUS), and these are discussed in detail later in this paper.

### 4.6. Anti-GBM Glomerulonephritis

Anti-glomerular basement membrane (GBM) is a rare life-threatening autoimmune disease, caused by IgG autoantibodies against alpha 3 NC1 domain of collagen IV of the GBM. Antibody binding to the GBM leads to injury characterized by strong complement activation, leukocyte infiltration, and proteinuria; leading to crescent formation, scarring, and, frequently, end-stage renal disease (ESRD). Evidence of complement pathogenic role comes from detection of complement components MBL, C1q, factor B (fB), properdin, C3d/C4d, and C5b-9 in GBM, and circulating MAC levels that correlate with kidney injury severity [48]. Local complement activation produces C3a- and C5a-mediated inflammation, as well as MAC-dependent sublytic activation of glomerular cells, which together enhance inflammation and extracellular matrix formation [49]. Pathways of complement activation in anti-GBM disease have been studied in murine models by injection of heterologous antibodies against GBM, where C3 and C4 deficiency prevented full manifestation of renal disease [46,50,51]. This evidence supports involvement of, at least, both classical and alternative pathways in anti-GBM disease.

### 4.7. ANCA Induced Renal Vasculitis

Antineutrophil cytoplasmic antibody (ANCA) associated vasculitides commonly target the kidney, with abundant complement component deposition in vessels and glomeruli without immunoglobulin (pauci-immune). Current research supports that vascular injury is due to cytokine-primed neutrophils displaying surface ANCA-binding antigens (myeloperoxidase and proteinase-3) that undergo degranulation while simultaneously activating alternative complement pathways which potentiates neutrophil recruitment via C5a [8]. C5a generation functions as an amplification loop for ANCA-mediated neutrophil activation, eventually culminating in the severe necrotizing inflammation of the vessel walls. Studies in animal models have also shown complement contributes to pathogenesis, and agents blocking C5 cleavage/C5a signaling or C5 and fB deficiency themselves are protective; conversely, preventing MAC formation is ineffective, supporting the importance of C5a in ANCA-mediated pathogenesis [52,53]. A recent trial of patients with ANCA vasculitis compared the use of rituximab/cyclophosphamide in addition to either placebo and high-dose steroids, avacopan (C5aR antagonist) plus reduced dose steroids, or avacopan and no steroids. Regimens with low or absent steroids were non-inferior to traditional regimens; this illustrates complement’s essential role in ANCA vasculitis and suggests C5aR antagonism as a feasible alternative in patients where steroids are contraindicated [54].

### 4.8. Lupus Nephritis

Systemic lupus erythematosus (SLE) is an autoimmune disorder characterized by antibodies to self-antigens (e.g., anti-nuclear) leading to the formation of immune complexes that deposit in target organs. Lupus nephritis (LN) is one severe complication of SLE. Up to 50% of patients with SLE have clinically evident kidney disease at presentation, and up to 75% develop it during the course of the disease [55]. The hallmark of renal pathology is simultaneous glomerular deposition of IgG, IgM, IgA, C4, and C3, referring to the poker hand ranking, the “full house pattern” [55]. Complement deposition is not merely a biomarker of LN, as it mediates direct glomerular injury: Immune complex-mediated classical pathway plays a key pathogenic role through both intra capillary generation of neutrophil and macrophage chemotactic factors (class II–IV) and formation of MAC (class V) [56]. Circulating C3 and C4 levels are reduced in more than 90% of patients with diffuse proliferative LN and their decline often reflects a worsening in disease activity [57]. Extensive data from animal models also indicate a significant role for alternative pathway activation: Deletion of regulators (i.e., fH) or activators (i.e., fB and fD) worsen or ameliorate, respectively, experimental LN [58,59,60]. In humans, plasma Bb levels (but not C3) are associated with LN outcome and strongly correlated with MAC levels. In addition, Bb co-localized with MAC in the glomeruli with LN, overall supporting the concept that activation of MAC in LN reflects alternative pathway activation [57]. Experimental LN can be prevented by blockade of all complement pathways through the administration of CR2-*Crry* fusion protein [61]. Data also show that the disease severity can be ameliorated by C5aR blockade [62] or anti-C5 mAb [63], which suggests the potential clinical relevance of complement pathway intervention. A phase 1 human trial with eculizumab (anti-C5) suggested preliminary efficacy, but the treatment period was too short to draw definitive conclusions [64]. The complement system seems to have a paradoxical role in SLE: genetically determined complement deficiencies or development of anti-complement antibodies involving components of the classical pathway (anti-C1q or C1-INH) [65], are strong risk factors. Susceptibility is likely due to a defective clearance of nuclear antigens released by injured and apoptotic cells since experimental studies have shown that such deficiencies lead to autoantibody production and glomerular injury [66].

## 5. Disease with Complement Activation in the Absence of Detectable Serum Antibodies

### 5.1. Atypical Hemolytic Uremic Syndrome

Hemolytic uremic syndrome (HUS) is defined by the triad of mechanical hemolytic anemia, thrombocytopenia and acute kidney injury. Renal pathology shows typically diffuse fibrin thrombi, endothelial swelling and capillary lumens narrowed/collapsed (acute features), or reduplication of GMB, mesangiolysis, and vessels recanalization (chronic phase). Typical forms of HUS are related to infection by Shiga toxin (Stx) producing *Escherichia coli* (STEC), while aHUS) is a condition due to defects in alternative complement activation. It has been associated with a predisposing genotype, usually an inherited heterozygous mutation [67,68], rather than an acquired mutation or loss of complement proteins (e.g., fI, fH, MCP).

The first identified mutant gene encodes for fH [28], the most important alternative pathway regulator in plasma and on cell surfaces. Subsequently, over 100 mutations were identified and most commonly lead to normal levels of a protein that is unable to bind and regulate complement components on endothelial cells [69].

Most complement genes mutations associated with aHUS result in an altered cell surface regulation: MCP mutation and fI mutation prevent effective degradation of C3 convertase. Although rarer, factors C3/C3b or fB gain of function mutations have been described [28]. Formation of blocking antibodies direct against fH is also another possible pathogenic mechanism [46,68]. 3–5% of patients with aHUS also carry heterozygous mutation of thrombomodulin (THBD), a molecule that normally enhances fI function [70]. As discussed below, complement targeting therapies have been extremely effective in treating this condition.

### 5.2. C3 Nephropathy

C3 nephropathy (C3N) is a rare nephritic disease, with a poor long-term outcome. The membranoproliferative pattern is the most common (not unique) histological presentation of C3 nephropathy and is further divided in the two entities of: Dense deposit disease (DDD), with typical ultrastructural evidence of intramembranous highly electron-dense osmophilic deposits with or without IgG and C3 on immunofluorescence, and C3 glomerulonephritis (C3GN) diagnosed with C3 positive on IF while all others immunoglobulins are negative [71,72]. Low serum C3 and glomerular deposits of C3 are emblematic of alternative pathway dysregulation.

A major subset of C3 glomerulopathies arises from C3Nef autoantibodies (present in 40% of C3GN and 80% of DDD) stabilizing C3 convertases against complement regulatory proteins (CRegs), or from other antibodies (former anti-fB, anti-fH) targeting directly CRegs [47]. C3 glomerulopathy can be due to genetic missense or non-sense mutations, affecting genes that encode for complement components or regulators [46,73]. The most important seems to involve fH, fI, and CFHR proteins with loss of function [74]; C3 mutation with gain of function and resistance to fH is uncommon [75] and when C5 genes are affected a less severe form of GN occurs [76]. fH/fI deficiency/resistance play a critical role in developing the disease because the GBM does not express CRegs and therefore relies on circulating ones (i.e., fH/fI) to prevent excessive local fluid phase AP activation.

Several cases of familiar C3 glomerulopathy have also been described when mutation of CFHR gene cluster occurs; recently an autosomal dominant inheritance among some Cypriote families has been described. In this nephropathy, CFHR5 has reduced affinity for surface-bound complement [77], the glomeruli present with C3GN features but C3 levels in the serum tend to be normal suggesting that improper complement activation occurs in the glomerulus rather than plasma [46,78,79,80,81].

## 6. Other Glomerular Diseases

### Focal Segmental Glomerulosclerosis

Focal segmental glomerulosclerosis (FSGS), is one of the leading causes of nephrotic syndrome in adults. Patients with non-nephrotic proteinuria have good prognosis and about 15% progress to ESRD over the course of 10 years, whereas 50% of patients with nephrotic-range proteinuria progress to ESRD over 5–10 years [82]. In patients with massive proteinuria (10–14 g/d), the course is malignant, resulting in ESRD by 2–3 years on average [82]. It is characterized by focal and segmental obliteration of glomerular capillary tufts, with an increase in matrix. The sources of podocyte damage are varied and not altogether known, but include circulating factors, genetic abnormalities, viral infection, and medications that produce common deleterious effects on podocytes. C3 and IgM glomerular deposition is typical, suggesting complement activation contributes to FSGS pathogenesis [83]. Sclerotic lesions are significantly higher in patients with C3 deposition combined with IgM; they have worse renal dysfunction and limited response to therapy [84]. Murine models of FSGS clarified that glomerular IgM deposits activate complement, suggesting that glomerular injury simultaneously increases classical pathway activation by natural IgM, which binds to injury associated epitopes, while also decreasing glomerular alternative complement regulating abilities [85]. Consistent with a pathogenic role for IgM, B cell absence in murine FSGS models prevents IgM deposition and albuminuria [85]. In humans, mutations in fH and C3 have been described in literature cases of biopsy documented FSGS [86] and FSGS patient urine and plasma are enriched with complement fragments C3a, C3b, Ba, Bb, C4a, sC5b-9 compared to samples from patients with other renal diseases [87].

## 7. Complement Inhibitory Drugs in Kidney Diseases

Identification of complement component contributions to renal pathogenesis recently spurred pharmaceutical industry efforts to therapeutically target complement 

Many available agents target terminal complement molecules and more proximal roles, such as opsonization, remains preserved. Even so, since these agents are immunosuppressants, they convey increased infection risk. More importantly, relevant to kidney diseases, many upstream elements (e.g., C3a, C5a, and C3b) contribute to pathogenesis and are not effectively targeted by available compounds. Currently, Food and Drug Administration (FDA) approved complement inhibitors include the monoclonal anti-C5 antibody Eculizumab, and Cynrize an inhibitor of fragment C1 (C1-INH) [88,89,90].

### 7.1. Eculizumab

Eculizumab is a humanized murine monoclonal antibody to complement C5 that acts on the terminal complement cascade preventing the formation of C5a, C5b, and C5b-9. The drug use has been approved by European Medicines Agency’s (EMA) and FDA for treatment of paroxysmal nocturnal hemoglobinuria (PNH) and aHUS [91]. The major side effect is predisposition to infections, especially from gram-negative bacteria; as such, all patients are advised to be immunized for *meningococcus* before receiving eculizumab. Eculizumab approval for aHUS treatment was given based on results from two prospective trials: one involving 17 aHUS patients with thrombocytopenia and the other with 20 aHUS patients requiring persistent plasma exchange (PE) [92]. Whole patients from these cohorts no longer required PE and 88% reached normal hematological values after median of 63 weeks of Eculizumab treatment. This medication has dramatically improved renal morbidity, with consistent decreases in ESRD risk, but its clinical use is still limited by uncertainty over patient selection, timing, and duration of treatment. An ongoing multi center single-arm trial is now testing the safety Eculizumab discontinuation in patients with aHUS (NCT02574403). Eculizumab has also been successfully used to prevent or treat recurrence of aHUS after kidney transplant [93,94,95], but appears ineffective in preventing delayed graft function (NCT01919346) [96] in sensitized kidney transplant recipients (NCT00670774, NCT01095887, NCT01327573) [97,98].

Eculizumab has successfully treated patients with DDD and C3GN highlighting its potential with these rare diseases. Treatment of six patients (three C3GN and three DDD) resulted in complete to partial remission in four patients at one year of follow-up [99]. This positive effect was limited to patients with crescentic rapidly progressive C3 glomerulopathy as opposed to a more insidious C3GN suggesting that it is specific to disease pathogenesis. Moreover, advantages are not seen so far in C3GN recurrence after kidney transplantation [100]. Eculizumab use in patients with glomerular diseases other than aHUS or C3GN/DDD is limited to case reports and of uncertain efficacy. The price of eculizumab is a factor that limits its use. Competitors have in clinical development both similar agents targeting C5 (e.g., Ravulizumab), as well as agents that affect complement component C5a (i.e., Avacoban).

### 7.2. C1 Inhibitor

Despite the potential advantages of terminal complement inhibition, these approaches may not be sufficient in conditions stemming from more proximal complement activation. Classical complement pathway activation occurs when C1q binds the Fc portion of antigen bound immunoglobulin. Preventing this process is C1-esterase inhibitor and its absence or mutated function produces the condition hereditary angioedema. *Cinryze*, a human serum derived C1 inhibitor (C1-INH) is FDA approved for treatment of hereditary angioedema. C1-INH does not have approved indications for kidney disease, but a phase I/II study was conducted on highly sensitized renal transplant recipients randomized to C1INH or placebo. Antibody mediated rejection was prevented in all 10 patients receiving C1INH group and 9/10 only receiving placebo. Efficacy and safety of C1INH for treatment of acute antibody mediated rejection in kidney transplantation is being evaluated in a randomized double-blind study of donor-sensitized kidney transplants recipients (NCT02052141, NCT02547220) [101]. Results may broaden its use to patients with antibody mediated rejection.

## 8. Conclusions

The complement system is a complex network of proteins that augment immune system function and, in many cases, contribute to kidney disease pathogenesis. Increasing research suggests that selective interventions to stop cascade activation can halt or even reverse renal disease. Ongoing research, both translational and in animal models, will help delineate which pathway(s), and at what level, intervention could be effective. Although infrequently a primary insult, common mutations affecting complement regulation synergize with other pathological features perpetuating inflammation and, ultimately, nephron loss. The advent of selective complement-targeting therapeutics offers the opportunity for new treatment strategies for renal disease, an area in desperate need of new options.

## Figures and Tables

**Table 1:** Summary of complement blocking agents.

Name.	Class	Pharmacodinamics	Disease	Status	Additional Info
*Eculizumab*	Humanized monoclonal antibody	Binds C5 preventing MAC generation	aHUS, DDD, C3GN	Available for use in PHN and aHUS	First USA FDA-approved among anti-complement drugs
*Ravulizumab* *ALXN1210*	Humanized monoclonal antibody	Binds C5 preventing MAC generation	aHUS	Phase III for PHN	Induces prolonged decease of C5 plasmatic levels allowing longer dosing intervals compared to Eculizumab
*Coversin*	Small dimension recombinant protein	Prevents cleavage of C5 into C5a/C5b by C5 convertase	aHUS	Phase II for PHN	Valid alternative for patients bearer of C5 molecule polymorphisms which interferes with correct binding of Eculizumab
*Avacopan* *CCX168*	Small dimension anti-inflammatory molecule	Inhibits selectively C5aR	aHUS ANCA-vasculitides	Phanse III for ANCA vasculitides. Phase II for aHUS	Effective replacing high-dose glucocorticoids in treating vasculitis
*CDX-1135*	C1R-based molecule	Inhibits CR1	DDD	Phase I for DDD	
*Mirococept* *APT070*	CR1-based molecule	Inhibits CR1	IRI in Tx	Phase I for DDD, C3GN	
*Cinryze*	C1 estarase	Inhibits CR1	Antibody-mediated rejection in renal transplant	Available for use in HAE. Phase III for prevention of DGF in cadaveric allograft	FDA approved for hereditary angioedema
*Narsoplimab* *OMS721*	Humanized monoclonal antibody	Binds the mannan-binding lectin-associated serinprotease-2	aHUS, TTP IgAN	Phase II for aHUS, IgA, LES, MN, C3G	Multi-dose administration is needed
*ACH-4471*	Small dimension molecule	Inhibits factor D	aHUS	Phase II for IC-MPGN, DDD, C3GN	Oral assumption with delivery advantage over intravenously infused agents

aHUS: Atypical hemolytic uremic syndrome, DDD: Dense deposit disease, C3GN: C3 glomerular disease, MAC: Membrane attack complex, ANCA: Antineutrophilic cystoplasmic antibody, IRI: Ischemia riper fusion injury, TTP: Thrombotic thrombocytopenia, IgAN: IgA Nephropathy, HAE: Hereditary angioedema, DGF: Delayed graft function.
